# Prevalence and Factors Associated With Using Complementary Medicine Among Adults With Type 2 Diabetes in Primary Healthcare Centers of Al-Ahsa, Eastern Saudi Arabia

**DOI:** 10.7759/cureus.67953

**Published:** 2024-08-27

**Authors:** Ali H Al-Luwaym, Abdulkareem J Al-Quwaidhi, Rahma B Al-Ghadeeb

**Affiliations:** 1 Preventive Medicine Department, Al-Ahsa Health Cluster, Ministry of Health, Al-Ahsa, SAU

**Keywords:** primary healthcare center, saudi arabia, prevalence, type 2 diabetes mellitus, alternative medicine, complementary medicine

## Abstract

Background and aim

Type 2 diabetes mellitus (T2DM) is a major health problem in Saudi Arabia. The use of complementary and alternative medicine (CAM) for the management of T2DM is increasing, but there is limited research on its safety and efficacy. This study aimed to investigate the prevalence and factors associated with CAM use among patients with T2DM attending primary healthcare centers in Al-Ahsa, Saudi Arabia.

Methods

A cross-sectional study was conducted between January and March 2024 to assess CAM use among T2DM patients in Al-Ahsa. Data were collected through telephone interviews using a validated questionnaire covering demographics, diabetes information, and CAM use. A total of 499 participants completed the questionnaire.

Results

CAM use was reported by 106 (21.2%) participants, with fenugreek (51, 48.1%), cinnamon (48, 45.3%), and blackseed (29, 27.4%) being the most common. Key factors associated with CAM use included residence in the Eastern or Northern Region, older age, having diabetic complications, and lower frequency of doctor visits. Only 24 (22.6%) participants consulted their doctor about CAM, and 83 (78.3%) believed that CAM could prevent diabetes progression.

Conclusion

This study provides insights into the use of CAM among T2DM patients in Al-Ahsa, Saudi Arabia. The findings highlight the need for larger-scale longitudinal research to better understand the patterns and potential benefits and/or harms of CAM use. Developing targeted interventions and promoting evidence-based integration of CAM into the healthcare system could optimize patient care and encourage patient-provider communication regarding these therapies.

## Introduction

The prevalence of type 2 diabetes mellitus (T2DM) has been steadily increasing worldwide, resulting in a significant global burden. According to the International Diabetes Federation (IDF), approximately 537 million adults were living with diabetes in 2021, and this number is projected to rise to 643 million by 2030 and 783 million by 2045 [1]. According to the IDF, the prevalence of diabetes (20-79 years) in Gulf Cooperation Council countries is significantly high, ranging from 11.8% in Oman to 25.5% in Kuwait in 2021 [2,3]. In Saudi Arabia, nearly one in six (17.7%) adults (aged 20-79) has T2DM, making it a significant public health issue for the country [4].

The terms “complementary medicine” or “alternative medicine” refer to a broad set of healthcare practices that are not part of that country’s own tradition or conventional medicine and are not fully integrated into the dominant healthcare system. These terms are used interchangeably with “traditional medicine” in some countries [5]. Complementary and alternative medicine (CAM) therapies are seeing greater use in the treatment of T2DM. The most utilized CAM modalities for diabetes are herbal remedies, dietary supplements, and mind-body practices [6].

Some of the prevalent over-the-counter CAMs taken by diabetics include cinnamon, garlic preparations, fenugreek, and multivitamin formulations [7]. The clinical efficacy and safety of several CAM therapies for diabetes continue to be evaluated. Furthermore, there is a widespread myth that “natural” always means “safe” and a common belief that remedies from a natural origin are harmless and carry no risk. However, some medicinal plants are toxic by nature. In addition, as with all medicines, herbal medicines are expected to have side effects. These unfavorable events associated with herbal products are accountable to problems of quality issues. Adverse events may also occur from the incorrect use of the species of medicinal plants, wrong doses, interactions with other medications, and the use of products contaminated with potentially harmful substances, such as toxic metals, disease-causing microorganisms, and chemical residues used in agriculture [8]. While some research studies reported that CAM therapies may help manage some diabetes symptoms, more research is still required to fully understand their safety and effectiveness [6].

CAM use among individuals with T2DM varies globally, influenced by cultural and socio-economic factors. In Jamaica, Owusu et al. found that 65% of the participants with T2DM used CAM, with significant associations between CAM use and both self-reported knowledge and acceptance of combining CAM with prescribed medications [9]. In Bangladesh, Rafi et al. reported a 35.2% CAM usage rate, primarily influenced by low income, the lack of formal education, and the longer duration of diabetes, with many patients believing that CAM aids in better diabetes control [10]. Mori et al. reported a 38.2% CAM usage in Japan, linking it to lower health-related quality of life [11].

In the Eastern Mediterranean Region, Naja et al. found a 38% prevalence of CAM use in Lebanon, significantly associated with marital status, longer diabetes duration, and diabetic complications [12]. In the United Arab Emirates (UAE), Radwan et al. observed a 39.3% usage, with associations to age, sex, employment, education, and health insurance, and folk foods were the most popular CAM type [13].

In Saudi Arabia, Yaghmour et al. conducted a community-based cross-sectional survey involving 1,290 participants. They found that 40.9% used CAM, with bitter apple (95.3%), cinnamon (64.6%), and ginger (55.1%) being the most common. CAM use was higher among older, married individuals, particularly females; those on hypoglycemic medications; and those with glycosylated hemoglobin (HbA1c) levels between 7% and 10%. It was also more prevalent among those with dyslipidemia, chronic diseases, and a family history of diabetes [14].

Aljulifi et al. examined CAM usage patterns among 332 adults with type 1 and type 2 diabetes in Riyadh at King Abdulaziz Medical City and reported a usage rate of 26%. Older and employed individuals were more likely to use CAM, with black cumin (42%), fenugreek (28%), myrrh (24%), frankincense (22%), cinnamon (15%), garlic (15%), and onion (15%) being the most frequently used herbs [15]. Similarly, Almogbel et al. reported a prevalence of 29.1% among 444 participants in Al-Qassim, identifying ginger, vitamins and minerals, and cinnamon as the most commonly used [16]. Abdullah et al. conducted a cross-sectional study among 350 diabetic patients in Taif City hospitals, finding a 33.7% CAM usage rate, with 87.3% not consulting their doctors beforehand. Bitter apple was used by all participants, while 66.1% used cinnamon, and 55.1% consumed ginger. In contrast to other studies, no significant associations were found between CAM use and sociodemographic factors [17].

Existing research studies in Saudi Arabia highlighted the prevalence of using CAM among diabetic patients [14-17]. However, these studies primarily relied on convenient sampling methods, which potentially limit their generalizability. Furthermore, no studies have been conducted in the Eastern Province, particularly Al-Ahsa, to explore the patterns of using CAM among diabetics compared to other regions. It is known that using CAM can potentially interact with conventional diabetes management approaches, especially when patients and healthcare professionals lack sufficient knowledge about CAM. Hence, our study aims to investigate the current landscape of CAM use among diabetic patients attending primary healthcare centers in Al-Ahsa. By measuring the prevalence of CAM use, identifying associated factors, and determining the types of CAM modalities utilized, our study provides useful data for stakeholders to develop appropriate measures and raise awareness regarding CAM use within this specific region.

## Materials and methods

We conducted a cross-sectional study to measure the prevalence and factors associated with using CAM among T2DM patients in Al-Ahsa, Saudi Arabia, between January and March 2024.

A total sample size of 742 subjects was estimated [18] to detect a prevalence of using CAM of 40.9% (14), a power of 95%, a margin of error of 5%, and a design effect of two. We used a proportionate stratified random sampling technique to select the study participants. Primary healthcare zones served as strata for our study. The participants were selected through systematic random sampling from each stratum.

We included adult Saudi patients living in the Al-Ahsa region, aged 18-85 years, and diagnosed with T2DM at least one year prior to the study. The participants were recruited from a database managed by the Population Health Management Unit (PHMU) in the Al-Ahsa Health Cluster, which contains comprehensive information on all diabetic patients receiving care at primary healthcare centers. The total number of Saudi T2DM patients was 43,452 distributed over four zones: Northern (11,396, 26.23%), Eastern (12,246, 28.18%), Middle (12,221, 28.13%), and Southern (7,589, 17.47%). We excluded non-Saudi patients, those younger than 18 years or older than 85 years, those diagnosed with T2DM less than one year ago, and those who did not agree to participate in the study.

The participants were contacted via telephone by trained public health data collectors. Data collectors underwent two face-to-face meetings to evaluate their communication abilities and comprehension of the questionnaire through mock interviews. Group discussions were conducted as necessary to address queries raised by data collectors about questionnaire items. The patients who met the inclusion criteria were introduced to the study’s objectives and invited to participate. Interested patients were assured that any collected information would be kept confidential and used exclusively for research purposes. The participants have the freedom to decline to answer any questions with which they do not feel comfortable. Each interview lasted for 15-20 minutes.

Data were collected through the utilization of an electronic multicomponent questionnaire, comprising a total of 42 questions. This validated questionnaire was used by previous studies to evaluate the use of CAM in diverse populations [12,19,20]. It encompasses three sections: demographic data, information related to diabetes, and CAM usage. The questionnaire was modified and translated into Arabic and validated by five bilingual experts (a diabetologist, two family physicians, public health specialists, and preventive medicine specialists). The questionnaire was translated into Arabic and electronically distributed to the experts. The experts were granted full autonomy to modify, exclude, or add questions as needed. Their feedback was subsequently collected and discussed with the authors to develop the final version of the questionnaire. A total of 499 participants completed the telephone-based interviews.

Statistical analysis

The collected data were entered into Statistical Package for Social Sciences (SPSS) software version 26 (IBM SPSS Statistics, Armonk, NY). All statistical methods used were two-tailed. Descriptive results were presented as frequency distributions and percentages for qualitative variables and as mean with standard deviation for quantitative variables. Graphs were constructed using Microsoft Excel software (Microsoft Corp., Redmond, WA). All relations were assessed using Pearson’s chi-squared test and the exact probability test for small frequency distributions. Multiple stepwise logistic regression was used to assess the most significant predictors for using complementary medicine among the study participants based on the adjusted odds ratio and its 95% confidence interval. A p-value of ≤0.05 was used to indicate statistical significance.

Ethical considerations

Formal approval was obtained from the Institutional Review Board at King Fahad Hospital-Al-Hofuf (approval number: 11-E-2023). Participation in the study was voluntary, with all participants being informed about the research objectives. Informed verbal consent was obtained from all participants, and the privacy and confidentiality of personal data were guaranteed.

## Results

A total of 499 eligible patients with T2DM were included. Patients’ ages ranged from 20 to 90 years old with a mean age of 65.1 ± 11.8 years old. Exactly 277 (55.5%) were males, and 411 (82.4%) were married. As for education, 175 (35.1%) had basic education, 131 (26.3%) had secondary education, and 103 (20.6%) were highly educated. A total of 151 (30.3%) were retired, 177 (35.5%) were housewives, 34 (6.8%) were not working, and 137 (27.5%) were working. Monthly income of less than 2,000 Saudi riyal (SR) was reported among 61 (12.2%); 93 (18.6%) reported 5,000-10,000 SR, but 201 (40.3%) refused to tell. Only 88 (17.6%) patients had health insurance (Table 1).

**Table 1:** Sociodemographic data of type 2 diabetic patients in primary healthcare centers in Al-Ahsa, Saudi Arabia (N = 499) SD: standard deviation

Sociodemographic data	N	%
Residence region		
Central	142	28.5%
Northern	129	25.9%
Eastern	137	27.5%
Southern	91	18.2%
Age in years		
<40	38	7.6%
40-50	117	23.4%
51-60	169	33.9%
>60	175	35.1%
Mean ± SD	65.1 ± 11.8	
Gender		
Male	277	55.5%
Female	222	44.5%
Marital status		
Single	18	3.6%
Married	411	82.4%
Divorced/widow	70	14.0%
Educational level		
Illiterate	90	18.0%
Basic education	175	35.1%
Secondary education	131	26.3%
High education	103	20.6%
Employment status		
Not working housewife	34 and 177	6.8% and 35.5%
Retired	151	30.3%
Working	137	27.5%
Household monthly income in Saudi riyal		
<2,000	61	12.2%
2,000-5,000	80	16.0%
5,001-10,000	93	18.6%
>10,000	64	12.8%
No need to tell	201	40.3%
Health insurance		
Yes	88	17.6%
No	411	82.4%

The reported prevalence of using CAM was around 21.2% (106/499). As for pattern, frequency, and reasons for using CAM among the study patients, 101 (95.3%) of the users used CAM in the previous year, and 83 (78.3%) of them are still using it. A total of 24 (22.6%) asked their doctors about the CAM therapies/products used. As for sources of information about CAM, family beliefs and traditions were the most reported (39/106, 36.8%), followed by the Internet, social media (38/106, 35.8%); friends’ suggestions (23/106, 21.7%); and health practitioner (6/106, 5.7%). Exactly 33 (31.1%) used CAM once a week, and 37 (34.9%) used two or more times per week. Exactly 83 (78.3%) expected the prevention of the progression of diabetes after using CAM. As for other reasons for using CAM other than diabetes mellitus (DM), 42 (39.6%) used it for providing energy, 18 (17%) for weight loss, and 10 (9.4%) for other reasons. Only four (3.8%) cases had side effects after using CAM. Considering users’ feelings after using CAM, the most reported were being in a good psychological condition (55/106, 51.9%), strengthening of the body (30/106, 28.3%), and the disappearance of several symptoms (24/106, 22.6%), but 30 (28.3%) had no change. A total of 58 (54.7%) will recommend the use of the CAM treatment to other diabetic patients (Table 2).

**Table 2:** Pattern, frequency, and reasons of using complementary and alternative medicine (CAM) among the study patients (N = 106)

Pattern of use		N	%
Have you used CAM in the previous year?	Yes	101	95.3%
	No	5	4.7%
Are you currently using CAM?	Yes	83	78.3%
	No	23	21.7%
Have you asked your doctor about the CAM therapies/products you used?	Yes	24	22.6%
	No	82	77.4%
How did you learn about CAM?	Family beliefs, traditions, etc.	39	36.8%
	Internet, social media, etc.	38	35.8%
	Friends’ suggestion	23	21.7%
	Health practitioner	6	5.7%
How often do you use CAM?	Daily	4	3.8%
	Once a week	33	31.1%
	Two or more times per week	37	34.9%
	Monthly	21	19.8%
	On need	11	10.4%
What did you expect when you started using CAM?	Prevention of the progression of diabetes	83	78.3%
	Complete cure of disease	7	6.6%
	Relief of pain	3	2.8%
	No expectations	13	12.3%
Do you use CAM for any other medical conditions or for your general health (other than your diabetes)?	Providing energy	42	39.6%
	Weight loss	18	17.0%
	Others	10	9.4%
	No	36	34.0%
Have you suffered from any side effect of CAM?	Yes	4	3.8%
	No	102	96.2%
What was your feeling after using CAM?	Being in a good psychological condition	55	51.9%
	Strengthening of the body	30	28.3%
	No change	30	28.3%
	Disappearance of several symptoms	24	22.6%
	Physically worse	5	4.7%
Would you recommend the use of the CAM treatment to other diabetic patients?	Yes	58	54.7%
	No	21	19.8%
	Not sure	27	25.5%

**Figure 1:**
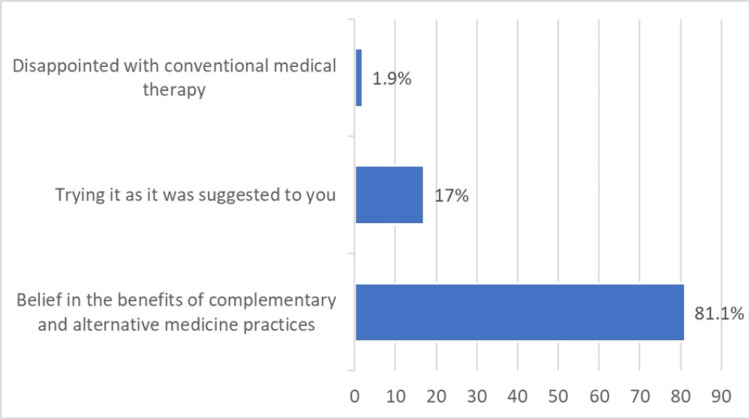
Reported reasons for using complementary and alternative medicine

The most reported reasons for using CAM were belief in the benefits of complementary and alternative medicine practices (86/106, 81.1%) and just to try (18/106, 17%) (Figure 1).

**Figure 2:**
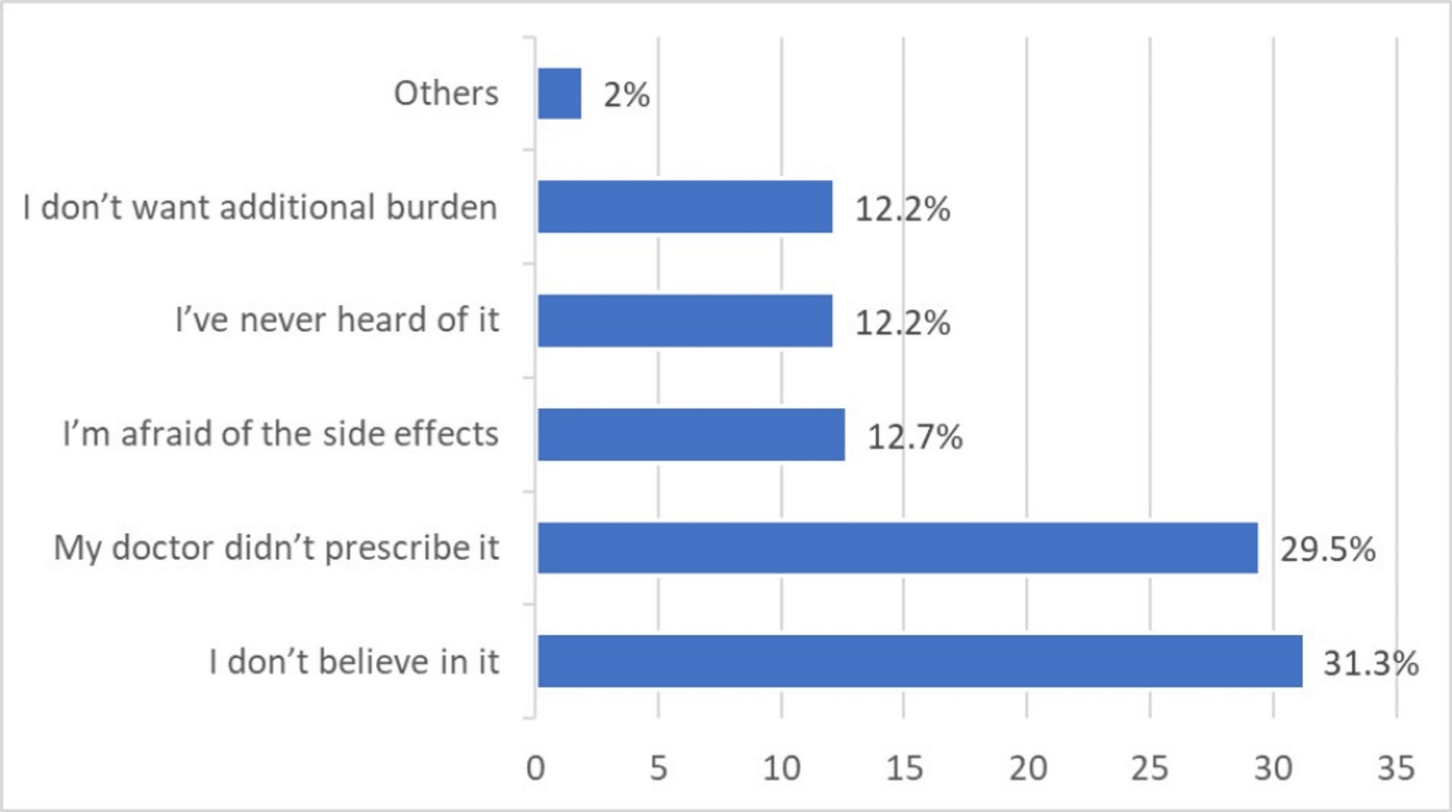
Reported reasons for avoiding complementary and alternative medicine

The most reported reason for avoiding CAM use was the lack of belief in its efficacy (31.3%). A significant proportion (29.5%) reported that their doctor had not prescribed CAM (Figure 2).

Table 3 shows the factors associated with CAM use among the study subjects. A total of 38 (27.7%) Eastern Region residents use CAM versus five (5.5%) Southern Region residents (P = 0.001). Also, 53 (25%) patients with diabetes complications used CAM in comparison to 53 (18.5%) of others without (P = 0.048). Likewise, 78 (24.1%) of those who visited their doctors used CAM less than once compared to more than once in three (8.1%) of those who visited their doctors (P = 0.046).

**Table 3:** Factors associated with complementary and alternative medicine (CAM) use among type 2 diabetic study patients in Al-Ahsa ^Exact probability test *P < 0.05 (significant)

Factors		Used CAM since diagnosed with diabetes?				P-value
		Yes		No		
		N	%	N	%	
Region	Central	29	20.4%	113	79.6%	0.001*
	Northern	34	26.4%	95	73.6%	
	Eastern	38	27.7%	99	72.3%	
	Southern	5	5.5%	86	94.5%	
Age in years	<40	6	15.8%	32	84.2%	0.147^
	40-50	33	28.2%	84	71.8%	
	50-60	36	21.3%	133	78.7%	
	>60	31	17.7%	144	82.3%	
Gender	Male	54	19.5%	223	80.5%	0.286
	Female	52	23.4%	170	76.6%	
Marital status	Single	6	33.3%	12	66.7%	0.436
	Married	86	20.9%	325	79.1%	
	Divorced/widow	14	20.0%	56	80.0%	
Educational level	Illiterate	20	22.2%	70	77.8%	0.632
	Basic education	42	24.0%	133	76.0%	
	Secondary education	24	18.3%	107	81.7%	
	High education	20	19.4%	83	80.6%	
Employment status	Not working	46	21.8%	165	78.2%	0.955
	Student/retired	32	21.2%	119	78.8%	
	Working	28	20.4%	109	79.6%	
Health insurance	Yes	17	19.3%	71	80.7%	0.627
	No	89	21.7%	322	78.3%	
Duration of diabetes in years	<5	33	20.9%	125	79.1%	0.505
	5-10	22	18.0%	100	82.0%	
	11-20	31	25.8%	89	74.2%	
	>20	20	20.2%	79	79.8%	
How do you manage your blood sugar?	Oral medication	83	20.6%	319	79.4%	0.472
	Insulin	33	22.3%	115	77.7%	
	Diet	14	26.4%	39	73.6%	
	Physical activity‎	14	29.2%	34	70.8%	
How often do you monitor your blood glucose level?	Daily	47	20.4%	183	79.6%	0.331
	Weekly	30	22.4%	104	77.6%	
	Monthly	12	31.6%	26	68.4%	
	Never	17	17.5%	80	82.5%	
Do you suffer from any complication of diabetes?	Yes	53	25.0%	159	75.0%	0.048*
	No	53	18.5%	234	81.5%	
Diabetes control	Poor	12	18.5%	53	81.5%	0.502
	Average	58	23.4%	190	76.6%	
	Good	36	19.4%	150	80.6%	
Do you adhere to your doctor’s recommendations?	Yes	83	21.3%	307	78.7%	0.574
	Sometimes	17	19.3%	71	80.7%	
	No	6	30.0%	14	70.0%	
Number of doctor visits per month	Less than once	78	24.1%	246	75.9%	0.046*^
	One time	25	18.1%	113	81.9%	
	More than once	3	8.1%	34	91.9%	

Multiple stepwise logistic regression was performed for predictors of CAM use among the study participants. Among all included factors, what is shown in the table were the most significant predictors of using CAM. CAM usage was significantly higher in the Eastern and Northern regions, with rates 6.6 and 6.2 times greater, respectively, than in other areas. Also, older age was associated with a 10% more likelihood of using CAM. Those with diabetic complications showed 40% more likelihood to use CAM, and patients with low visit frequency (less than once per month) to their doctors also showed 40% more likelihood of using CAM (Table 4).

**Table 4:** Multiple stepwise logistic regression for predictors of complementary and alternative medicine use among type 2 diabetic patients *P < 0.05 (significant) AOR, adjusted odds ratio; CI, confidence interval

Factors	P-value	AOR	95% CI	
			Lower	Upper
Region				
Central	0.036*	4.4	1.6	11.8
Northern	0.033*	6.2	2.3	16.4
Eastern	0.039*	6.6	2.5	17.5
Age in years	0.047*	1.1	1.1	3.9
Have diabetes complications	0.033*	1.4	1.1	2.2
Low visits (less than once)	0.042*	1.4	1.0	12.1

**Figure 3:**
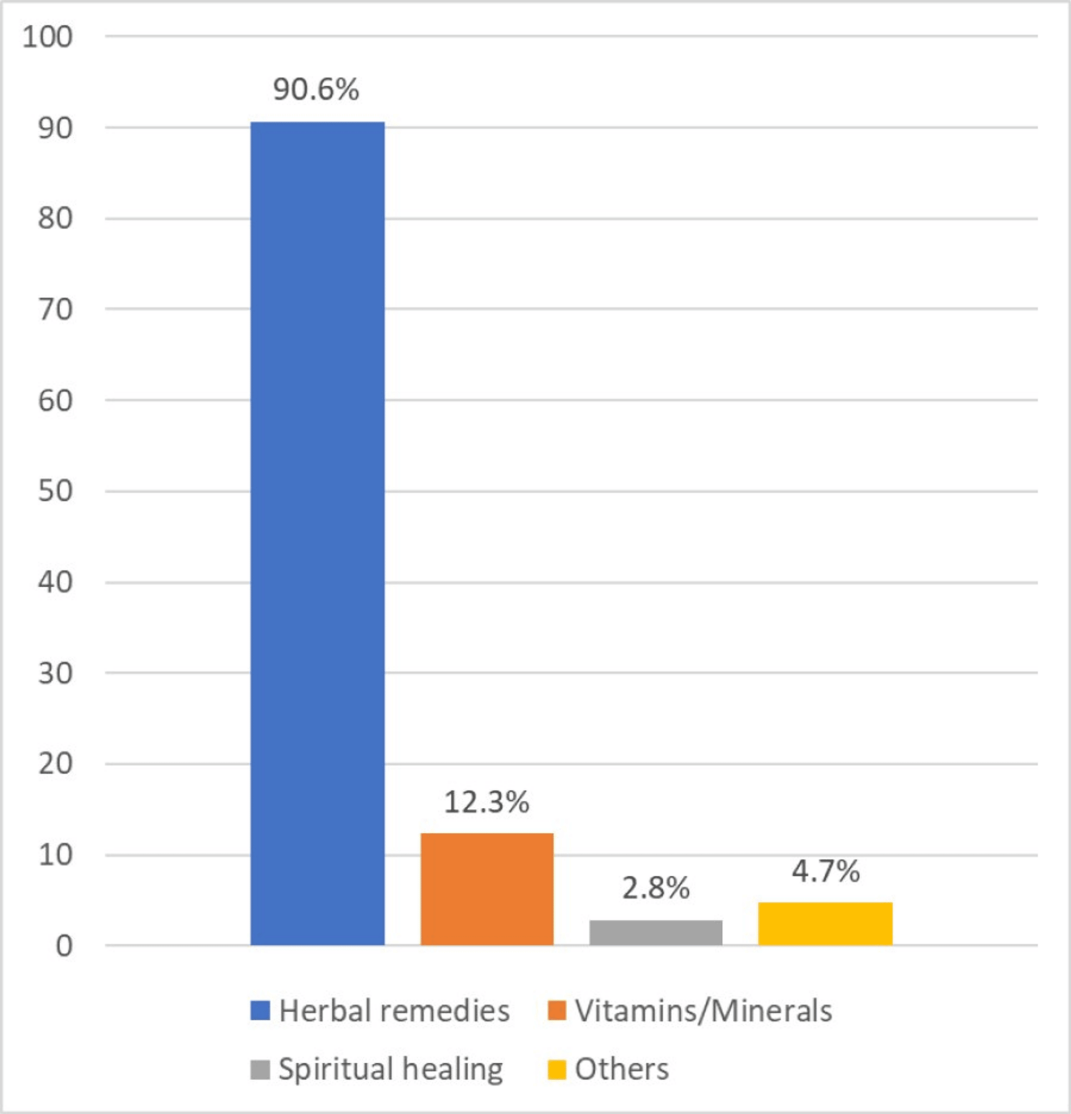
Reported common type of complementary and alternative medicine used

Herbal remedies emerged as the most widely utilized form of CAM, constituting 90.6% (96/106) of reported usage. Vitamins and minerals followed as a less common choice, accounting for 12.3% (13/106) of responses. Spiritual healing and other unspecified modalities represented smaller proportions of the sample, with 2.8% (3/106) and 4.7% (5/106), respectively (Figure 3).

**Figure 4:**
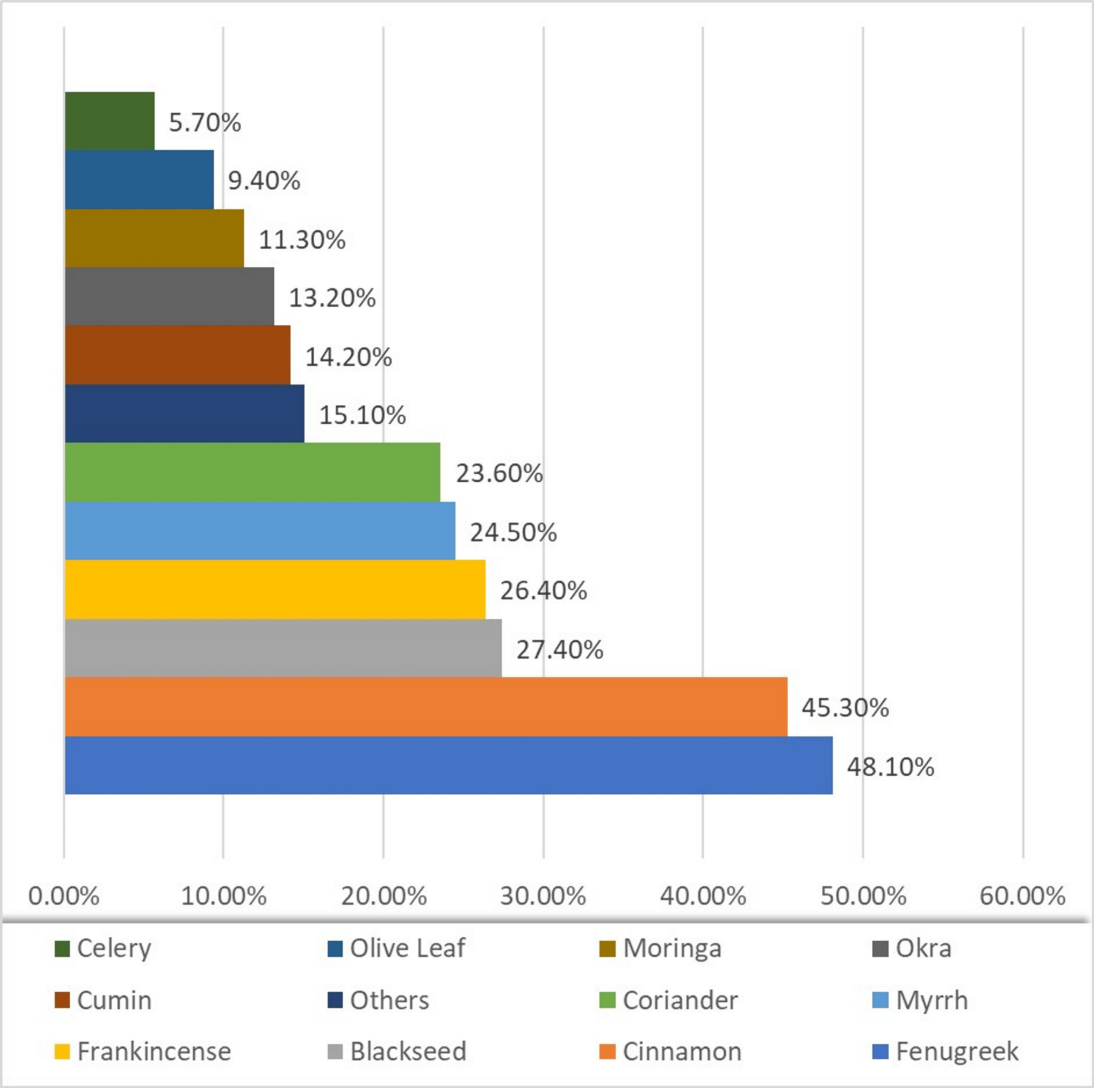
Complementary and alternative medicine items used for diabetes management

Fenugreek emerged as the most utilized CAM, accounting for 48.1% (51/106) of reported usage. Cinnamon followed, constituting 45.3% (48/106) of responses. Blackseeds, frankincense, myrrh, and coriander exhibited moderate usage rates at 27.4% (29/106), 26.4% (28/106), 24.5% (26/106), and 23.6% (25/106), respectively (Figure 4).

## Discussion

This study investigated the prevalence and factors associated with the use of CAM among patients with T2DM in Al-Ahsa, Kingdom of Saudi Arabia (KSA). Out of 499, 106 (21.2%) participants reported using CAM. Patients from the Eastern, Northern, and Central zones; those with existing diabetic complications and infrequent (less than once per month) doctor visits; and older adults were more likely to use CAM.

The prevalence reported by this study (21.2%) is considerably lower than other recent studies, such as that Owusu et al. [9] (60%) in Jamaica, Yaghmour et al. [14] (40.9%) in the KSA, Radwan et al. [13] (39.3%) in the UAE, Naja et al. [12] (38%) in Lebanon, and Rafi et al. [10] (35.2%) in Bangladesh. The lower prevalence in this study might reflect variations in sociocultural factors, healthcare access, patient awareness, or study methodology.

On the other hand, the results from several studies from Saudi Arabia appear to align with our findings. For example, the reported prevalence of using CAM was 26% in Riyadh, 29.1% in Al-Qassim, and 33.7% in Taif [15-17]. We found associations between CAM use and older age and the presence of diabetic complications, and this is consistent with results from other studies from Saudi Arabia [12-14]. This study identified fenugreek, cinnamon, and blackseed as the most common forms of CAM used by the participants. These forms of CAM were also reported in other studies [14-17]. Nevertheless, interestingly, some studies [14,17] reported bitter apple as a frequently used form of CAM, but in this study, none of the participants reported using the same form of CAM.

In this study, 82 (77.4%) participants did not report their use of CAM to their doctors. These results aligned with multiple studies ranging from 62.5% to 93.1% [12-15,17], which may be attributed to the fear of criticism and negative responses from healthcare providers. The research shows that many CAM users were referred to or encouraged to try CAM by their social network (family and friends) or social media. Such a finding is replicated in many previous studies [12-15,17].

This study sheds light on the widespread use of complementary and alternative medicine (CAM) among type 2 diabetics. It also explores factors associated with CAM use, giving healthcare providers valuable insights into patient characteristics. This knowledge can empower providers in both clinical and public health settings. By understanding the specific CAM modalities patients utilize, healthcare providers can have more informed discussions and recommendations. Furthermore, the study highlights the need for improved communication between patients and providers regarding CAM use. This can lead to better patient care and potentially improved health outcomes. The key strengths of this study include a relatively large sample size; the exploration of a previously uninvestigated area, Al-Ahsa; and the employment of probability sampling, which collectively enhance the credibility of the findings.

Limitations

Our study has some potential limitations. First, it relied mostly on self-reported information, which may result in some subjectivity and variations in responses. Second, phone-based interviews can be associated with a lack of visual cues, technical interruptions, and challenges to building rapport with the participants. Third, there were some missing data regarding recent HbA1c readings for some participants. Fourth, there was a possibility of social desirability bias in the participants’ responses. The participants may have been motivated to provide answers that they perceived as more socially acceptable or favorable, rather than accurately reflecting their true opinions, attitudes, or behaviors. Finally, the cross-sectional design of the study cannot indicate the temporal sequence of study variables. While including non-Saudi participants would enhance the generalizability of the findings, their relatively small proportion within the population utilizing these healthcare services suggests a potentially minor impact on the overall results.

Recommendations

Future research should consider longitudinal studies to comprehensively explore the dynamic nature of the relationship. A mixed-methods approach could provide deeper insights into the phenomenon. Additionally, expanding the sample size and including a diverse national representation would enhance the study’s power and generalizability. To optimize patient care and empower informed decision-making, this study recommends a multidimensional approach. Firstly, promoting open communication through educational materials for patients and communication training for healthcare providers can encourage patients to disclose CAM use. Secondly, further research on the most prevalent CAM modalities used for type 2 diabetes is crucial to establish valid resources and inform clinical practice. Thirdly, integrating CAM education into medical and public health curriculums can equip future healthcare professionals with the necessary knowledge for effective patient-provider dialogue. Finally, exploring options for establishing ethical guidelines and referral systems for qualified CAM practitioners can promote responsible CAM use within the larger healthcare system.

## Conclusions

Complementary and alternative medicine use is prevalent among patients with T2DM in Al-Ahsa, with herbal remedies such as fenugreek, cinnamon, and blackseed being the most common choices. Key factors influencing CAM use include geographic region, older age, the presence of diabetic complications, and the lower frequency of follow-up visits. Despite the high prevalence of CAM use, adverse effects are rarely reported. However, limitations such as social desirability and self-reporting should be considered in interpreting study findings. The study results highlight the need for larger longitudinal studies to further explore the patterns and causal relationships of CAM use in T2DM patients. This would allow for the development of more targeted interventions to optimize patient care and promote evidence-based integration of CAM into the healthcare system to improve patient outcomes and satisfaction.
